# The FUTUREPAIN study: Validating a questionnaire to predict the probability of having chronic pain 7-10 years into the future

**DOI:** 10.1371/journal.pone.0237508

**Published:** 2020-08-20

**Authors:** Timothy T. Brown, Woojung Lee

**Affiliations:** 1 School of Public Health, University of California, Berkeley, Berkeley, CA, United States of America; 2 School of Pharmacy, University of Washington, Washington, DC, United States of America; Monash University School of Public Health and Preventive Medicine, AUSTRALIA

## Abstract

**Objectives:**

The FUTUREPAIN study develops a short general-purpose questionnaire, based on the biopsychosocial model, to predict the probability of developing or maintaining moderate-to-severe chronic pain 7–10 years into the future.

**Methods:**

This is a retrospective cohort study. Two-thirds of participants in the National Survey of Midlife Development in the United States were randomly assigned to a training cohort used to train a predictive machine learning model based on the least absolute shrinkage and selection operator (LASSO) algorithm, which produces a model with minimal covariates. Out-of-sample predictions from this model were then estimated using the remaining one-third testing cohort to determine the area under the receiver operating characteristic curve (AUROC). An optimal cut-point that maximized sensitivity and specificity was determined.

**Results:**

The LASSO model using 82 variables in the training cohort, yielded an 18-variable model with an out-of-sample AUROC of 0.85 (95% Confidence Interval (CI): 0.80, 0.91) in the testing cohort. The sum of sensitivity (0.88) and specificity (0.76) was maximized at a cut-point of 17 (95% CI: 15, 18) on a 0–100 scale where the AUROC was 0.82.

**Discussion:**

We developed a short general-purpose questionnaire that predicts the probability of an adult having moderate-to-severe chronic pain in 7-to-10 years. It has diagnostic ability greater than 80% and can be used regardless of whether a patient is currently experiencing chronic pain. Knowing which patients are likely to have moderate-to-severe chronic pain in the future allows clinicians to target preventive treatment.

## Introduction

According to the Institute of Medicine, chronic pain is the most prevalent, disabling, and expensive health condition in the US [1]. Chronic pain affects 30.7% of adults (with 32% of these experiencing severe pain) annually costing $261–300 billion in healthcare expenditures and $299–335 billion in lost productivity [2, 3]. Combining these suggests national costs of approximately $560–635 billion, exceeding the cost of cancer, heart disease, and diabetes combined [4]. While mild chronic pain is a nuisance, individuals with moderate-to-severe chronic pain not only suffer interference in their daily activities, but are more likely to experience psychological problems [5]. Thus, moderate-to-severe chronic pain is the focus of this study.

Despite the burden of moderate-to-severe chronic pain on individuals and society, interventions to prevent and treat moderate-to-severe chronic pain are often inadequate or inaccessible, resulting in calls for coordinated efforts to transform approaches to preventing and treating chronic pain [1]. In 2014, The American Pain Society released its Pain Research Agenda for the 21^st^ Century [4]. It emphasized that one of the essential prerequisites to making advances in the prevention and management of chronic pain is to identify the risk factors that contribute to chronic pain, calling for a comprehensive approach that considers chronic pain not merely a physical symptom, but a condition influenced by biological, psychological, and social factors [6–8].

The biopsychosocial model has been developed to be inclusive of all categories of factors that influence the onset and maintenance of chronic pain [7]. Over the last decade, research has made considerable strides in understanding the determinants of chronic pain. Risk factors include sociodemographic factors [3, 9], adverse childhood experiences [10–14], personality traits [15–17], psychological factors [18–21], and physical factors [22–25].

Nevertheless, it is challenging for clinicians to predict who is likely to experience the onset or maintenance of moderate-to-severe chronic pain [3, 26]. For identified risk factors to have practical significance in identifying individuals who are at risk of developing or continuing to have moderate-to-severe chronic pain, concise and accurate screening tools must be established.

To date, a number of pain questionnaires have been developed and are being used by practitioners. However, their application has been limited to patients who already have pain symptoms, with a focus on predicting whether current acute pain will become chronic pain. For example, the Örebro Musculoskeletal Pain Questionnaire (OMPG) predicts the risk of long-term disability and sick leave after musculoskeletal injury [27]. Similarly, other questionnaires such as the STarT Back Tool (SBT), the Acute Low Back Pain Screening Questionnaire (ALBPSQ), and the Vermont Disability Prediction Questionnaire (VDPQ) were developed to stratify patients with acute low back pain into low, medium, or high-risk categories with respect to developing chronic back pain [28–31].

These questionnaires are of great value, but to our knowledge no single screening tool has been developed to date that can be used to predict new-onset moderate-to-severe chronic pain *or* the maintenance of chronic pain, depending on whether or not an adult is currently experiencing chronic pain. More timely prevention will become possible if we can predict who has a high probability of developing moderate-to-severe chronic pain years before moderate-to-severe chronic pain develops. Similarly, directing specific patients into self-management options that focus on lowering the probability that existing chronic pain will be maintained will also become possible if we know which patients with chronic pain are likely to experience moderate-to-severe chronic pain into the future.

Thus, the aim of this study is to establish and validate a parsimonious questionnaire that accurately predicts who will have moderate-to-severe chronic pain 7-to-10 years into the future, regardless of current pain status. Ours is the first prediction tool to enable clinicians to predict the probability of future moderate-to-severe chronic pain in adult populations with and without chronic pain.

## Material and methods

### Data

This retrospective cohort study uses data from the National Survey of Midlife Development in the United States (MIDUS) [32]. The MIDUS is comprised of a nationally representative sample of adults, a sample of the siblings of these adults, a national sample of twins, and a metropolitan sample. Participants were asked to provide information on their health status, sociodemographic factors, lifestyles, social responsibilities and psychological well-being via telephone interviews and mail questionnaires. Two longitudinal follow-ups of the original MIDUS samples (1995–1996) were performed in 2004–2006 (MIDUS II) and 2013–2014 (MIDUS III) [33–35]. During these follow-ups, data collection largely repeated baseline assessment but also included additional questions on selected topics. We combined data across the various samples from the first (MIDUS), second (MIDUS II), and third waves (MIDUS III). In addition, we included data from the biomarker project (MIDUS II) in order to include additional psychological and clinical variables. The Biomarker Project is a separate, but related, study that aims to add broad biological assessments to a subsample of MIDUS II respondents [36].

**Fig 1:**
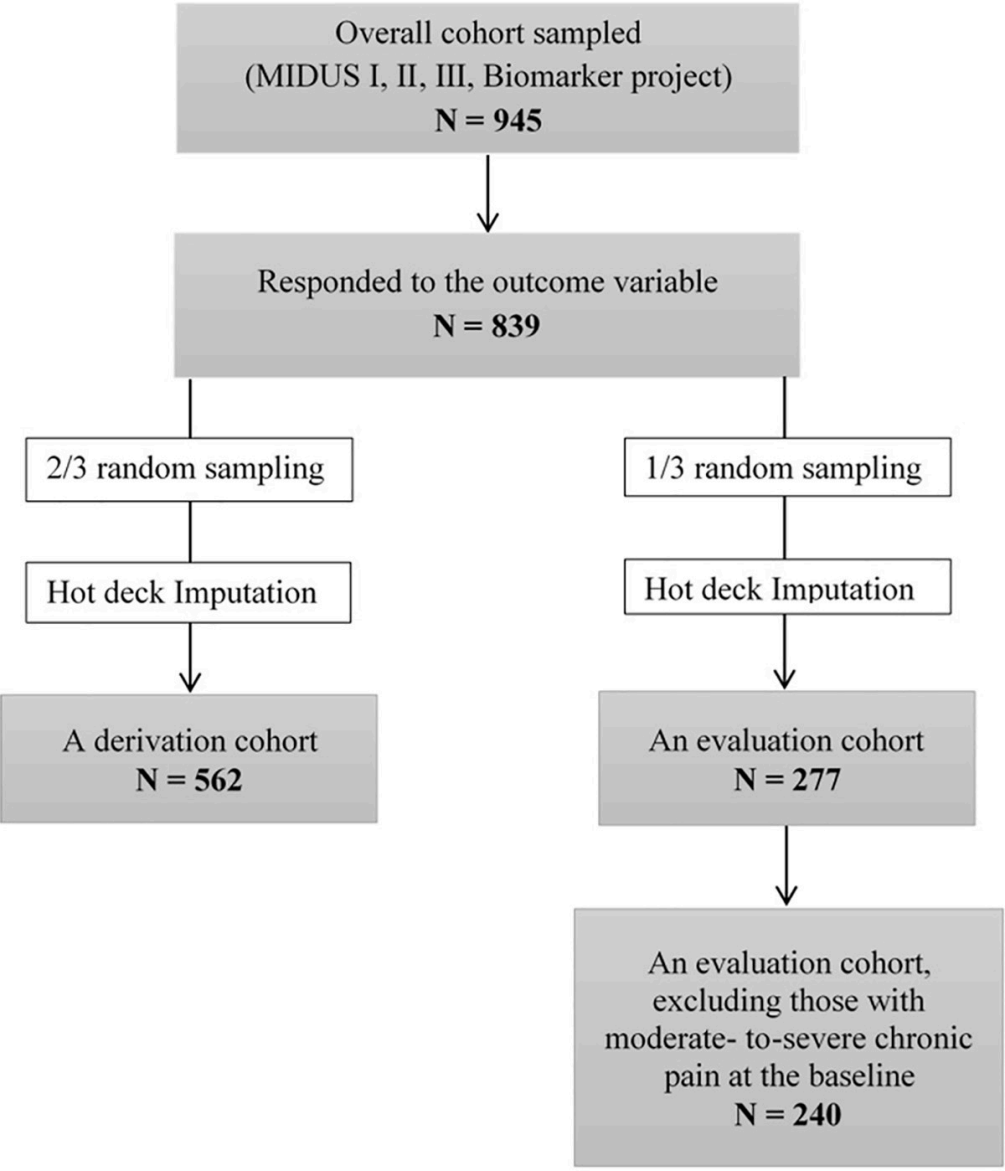
Flow chart of participants in the study.

Of 945 who participated in MIDUS I, II, III, and the Biomarker Project, 106 participants were excluded from the analysis due to missing outcome data (Fig 1). Of 839 participants who remained, two-thirds were randomly sampled and assigned to a training cohort and the remaining one-third was assigned to a testing cohort (Fig 1). Hot deck imputation was used to impute missing values in the independent variables [37]. Statistical differences in participants’ sociodemographic factors between imputed and non-imputed data were assessed using *Z*-tests and *t*-tests for comparing proportions and means, respectively.

### Dependent variable

The outcome of interest was having moderate-to-severe chronic pain. To construct this outcome, we combined each participant’s answer to a screening question with their answers to the five questions that make up the Brief Pain Inventory (BPI) interference scale, both of which are included in MIDUS III. First, participants were asked the screening question, “Do you have chronic pain, that is, do you have pain that persists beyond the time of normal healing and has lasted anywhere from a few months to many years?” For those who answered yes, the severity of chronic pain was determined using the BPI interference scale, a valid and reliable questionnaire for measuring the severity of chronic pain [38, 39]. The five questions included in this scale asked participants the extent to which pain interfered with the respondent’s daily activities within the last week across the following areas: general activity, relations with other people, sleep, mood, and enjoyment of life. The response options range from 0–10 for each area, with a score of 0 indicating chronic pain is not significant enough to interfere with the activity, and 10 indicating the activity was completely interfered with. We calculated the average score of the five answers and constructed a binary outcome variable indicating moderate-to-severe chronic pain by assigning 1 to those whose average score is equal to or greater than 4 and 0 otherwise [40, 41].

### Independent variables

Potential risk factors for chronic pain were selected based on a review of epidemiological studies of chronic pain, theoretical relevance, and the availability of potential risk factors in our dataset. We included sociodemographic factors, adverse childhood experiences, life problems, major life traumas, psychological factors, personality traits, clinical factors, and baseline pain status. Specific information on the items and scales contained in the MIDUS are publicly available [42].

Sociodemographic factors were collected from the MIDUS II. These include age, sex, race, education, marital status, having children, employment status, annual income, and self-reported health status.

We generated 9 variables on adverse childhood experiences based on the Adverse Childhood Experience (ACEs) Questionnaire that assesses physical, emotional, and sexual abuse; parental neglect; and household dysfunction [43]. Independent variables that correspond to the ACEs items were obtained from MIDUS I, II, and III. We matched ACEs items and independent variables as precisely as possible by either choosing the items with the most relevant content or by combining multiple items into one variable. All ordinal variables were converted to binary variables in accordance with the ACEs Questionnaire.

We selected 7 measures of life problems (daily discrimination, lifetime discrimination, current work situation, family strain, friend strain, marital risk, and current rating of financial situation) defined as stressful events that frequently occur throughout life; and 14 major life traumas (flunked out of school, fired from job, no job for a long time, parents divorced, partner engaged in infidelity, in-law difficulties, recent death in the family, lost home to fire/flood, sexual assault, serious legal difficulties/prison, jail detention, bankruptcy, other financial loss, military combat) defined as extraordinarily stressful but infrequent life events. Four of these variables are measured using valid and reliable scales with the same names: family strain [44–46], friendship strain [45, 46], daily discrimination [47], and lifetime discrimination [47]. Death(s) in the family within the last five years was generated using a series of questions asking whether and when a participant’s parents, siblings, or children had died. Other variables were measured using single items, with either dichotomous or ordinal responses.

Fourteen variables measuring psychological factors, described below, were chosen based on their use in prior studies identifying the variables as potential psychological risk factors for negative health outcomes [48, 49]. The first five of the fourteen variables are five subscales that represent the three categories of symptoms of anxiety and depression: negative affect (NA), positive affect (PA), and physiological hyper-arousal (PH)—based on the tripartite model of depression and anxiety as expressed by the Mood and Anxiety Symptom Questionnaire (MASQ) [50, 51]. With regard to NA, we used the following two MASQ subscales of “general distress depressive symptoms,” a measure of depression, and “general distress anxious symptoms,” a measure of anxiety [50]. With regard to PA, we used the two subscales of the MASQ anhedonic depression scale: “loss of interest” assessing depressive symptoms and “high positive affect” consisting of reverse-scored items related to pleasant emotions [52]. The final MASQ subscale “anxious arousal symptoms” was used to measure PH [53].

The sixth of the fourteen variables is the Center for Epidemiologic Studies Depression Scale (CES-D) that assess the symptomatology of depression [54]. The purpose of including a measure of depressive symptoms in addition to the Anhedonic Depression scale was to choose the scale that differentially is a better predictor of moderate-to-severe chronic pain.

The seventh through tenth of the fourteen variables are the four subscales of the Spielberger State-Trait Anger Expression Inventory that assess experience and expression of anger [55]. To assess expression of anger, three subscales, “Anger Expression-In”, “Anger Expression-Out”, and “Anger Expression-Control”, were used. The subscale “Trait Anger” was used to measure experiencing anger.

The eleventh of the fourteen variables is the Kessler K6. The K6 is a valid and reliable measure of non-specific psychological distress based on six questions [56].

Lastly, the twelfth through fourteenth of the fourteen variables are three major coping measures: personal religiosity and spirituality daily guidance and coping [57, 58], problem-focused coping [58, 59], and emotion-focused coping [58, 59]. Personal religiosity and spirituality daily guidance and coping, a two-variable scale [57, 58], assesses the extent to which an individual seeks spiritual or religious support when in a stressful or decision-making situation [57, 60]. Problem-focused coping, part of the COPE inventory [59] is made up of three subscales measuring “positive reinterpretation and growth”, “active coping”, and “planning” [58, 61]. The subscales of the COPE inventory “focus on and venting of emotion”, “denial”, and “behavioral disengagement” are part of a larger combined scale measuring emotion-focused coping [58, 59, 61].

To measure personality factors, which we separate from the above psychological factors, we used the valid and reliable Big Five personality scales obtained from MIDUS II to measure the following personality traits: conscientiousness, agreeableness, neuroticism, openness, and extraversion [62]. In addition, the measure somatic amplification [58], which is known to be predictive of chronic pain, was included [63, 64].

Eight clinical factors were chosen based on studies of clinical risk factors for chronic pain, including body mass index, number of vehicle injuries, number of broken bones, number of surgeries, number of head injuries, number of joint injuries, sleep quality, and low pain tolerance [23, 65–69]. Sleep quality was self-rated from 1 to 4, looking back at the past month with 1 being very good and 4 being very bad. Participants also self-reported if they have low pain tolerance from 1 to 4, with 1 being not at all true and 4 being extremely true.

Finally, chronic pain and acute pain from MIDUS II were included. A binary variable of baseline chronic pain was generated based on a single item that asks if respondents are experiencing pain that persists beyond the time of normal healing and has lasted anywhere from a few months to many years. A binary variable of baseline acute pain was generated using 4 items asking how often one has experienced headache, backache, joint aches, and extremities aches in the past 30 days. Among these individuals, those who also reported no chronic pain, and whose pain happens “several times a week” or “almost every day” for at least one of the four kinds of pain above were assumed to be experiencing acute pain.

### Statistical analysis

To avoid bias, we did not develop multiple models, instead we included all of the variables identified above in a single model. The final questionnaire was then determined by our statistical modeling procedure: we applied the least absolute shrinkage and selection operator (LASSO) algorithm, a machine learning approach to variable selection in which lambda, a parameter that specifies the penalty placed on coefficients, is determined statistically by internal cross-validation [70]. This approach is completely automated and no variables were removed based on our personal judgement. The LASSO model was trained on a 67% random draw of the data, the training sample, and then evaluated using the hold-out 33% of the data, the testing sample, which produced a final area under the receiver operating characteristic curve (AUROC) based on the out-of-sample prediction. We additionally calculated an AUROC in a hold-out sample that omitted those who had moderate-to-severe chronic pain as of MIDUS II in order to assess how well the model predicts the onset of moderate-to-severe chronic pain among those without moderate-to-severe chronic pain at baseline. Based on the results of the full testing sample, we determined the cut-off score, based on Youden’s *J* index, that indicates sufficient risk to initiate preventive therapies [71, 72]. All statistical analyses were conducted using Stata 15 (StataCorp LLC). This study uses unidentifiable and publicly-available data and is not considered to be human subjects research by the Committee for the Protection of Human Subjects at the University of California, Berkeley.

## Results

### Cohort description

Table 1 presents selected descriptive statistics of the training cohort and the testing cohort. Among the training cohort, 34% experienced chronic pain at baseline, while 15% experienced moderate-to-severe chronic pain 7–10 years later. 56% were female and 93% were Caucasian [73]. Their mean age was 54.4 (SD = 10.9, range = 34–81). *Z* tests (for proportions) and *t* tests (for means) did not indicate statistical differences in the participants’ characteristics in the imputed versus non-imputed cohorts. Table 1 also shows that the testing cohort is not statistically different from the training cohort with respect to participant characteristics.

**Table 1:** Selected characteristics of training and testing cohorts.

Characteristics	Proportion or Mean (SD^a^)		*P* Value^b^
	Training Cohort (n = 562)	Testing Cohort (n = 277)	Difference between Training and Testing
**Age (MIDUS II)**^c^			
20–34	0.00	0.01	0.96
35–44	0.21	0.18	0.69
45–54	0.30	0.32	0.76
55–64	0.26	0.33	0.32
65+	0.22	0.16	0.35
**Gender (MIDUS II)**^c^			
Female	0.55	0.56	0.91
**Race (MIDUS II)**^c^			
White	0.93	0.93	0.99
Black	0.03	0.01	0.83
Others	0.04	0.05	0.82
**Education (MIDUS II)**^c^			
Some high school or lower	0.03	0.02	0.91
High school graduate	0.19	0.22	0.65
Some college	0.27	0.31	0.64
College graduate	0.25	0.23	0.77
Graduate school or higher	0.25	0.22	0.61
**Marital status (MIDUS III)**^c^			
Married	0.72	0.77	0.46
Separated	0.02	0.01	0.91
Divorced	0.12	0.12	0.96
Widowed	0.05	0.03	0.76
Never married	0.09	0.07	0.73
**Children (MIDUS II)**^c^			
No children	0.15	0.11	0.64
**Employment status**			
Employed	0.71 (0.04)	0.72 (0.06)	0.86
Unemployed	0.02 (0.04)	0.02 (0.06)	0.96
Not in workforce	0.35 (0.04)	0.32 (0.06)	0.68
**Annual income**^d^	45589.41(1736.21)	45857.4 (2372,4)	0.92
**Self-reported physical health status (MIDUS II)**^c^			
Excellent	0.22	0.21	0.92
Very good	0.44	0.43	0.93
Good	0.27	0.29	0.86
Fair	0.06	0.06	0.99
Poor	0.01	0.01	0.98
**Baseline pain status (MIDUS II)**^c^			
Acute pain	0.26	0.25	0.88
Chronic pain	0.34	0.33	0.90
**Other variables included in the prediction model (MIDUS II)**			
Parental abuse^c^	0.37	0.38	0.86
Death in family^c^	0.17	0.21	0.58
Lost home^c^	0.04	0.03	0.87
Financial or property loss^c^	0.04	0.06	0.84
Loss of Interest^d^	11.77 (3.99)	11.33 (3.39)	0.11
Anxious arousal^d^	21.58 (5.19)	20.99 (4.35)	0.10
Kessler K6^d^	1.45 (0.50)	1.46 (0.54)	0.75
Trait Anger^d^	23.57 (5.17)	23.63 (5.40)	0.87
Religious coping^d^	5.71 (2.16)	5.74 (2.11)	0.85
Body mass index^d^	27.58 (5.26)	27.91 (5.63)	0.40
Sleep quality^d^	1.94 (0.65)	1.97 (0.70)	0.43
Number of surgeries^d^	3.54 (2.55)	3.52 (2.64)	0.92
Number of vehicle accidents with injuries^d^	0.47 (0.75)	0.40 (0.63)	0.20
Number of head injuries^d^	0.33 (0.33)	0.40 (0.71)	0.15
Number of joint injuries^d^	0.70 (0.89)	0.61 (0.86)	0.13
Low pain tolerance^d^	1.78 (0.04)	1.80 (0.05)	0.80
**Outcome variable (MIDUS III)**^c^			
Moderate-to-severe chronic pain	0.13	0.16	0.75

^a^Standard deviations were not calculated for binary variables

^b^*Z* tests were used to compare proportions and unpaired *t*-tests were used to compare means from two independent samples.

^c^Proportions

^d^Means. See Table 2 for a complete list of the range of each variable.

### Predictive performance of prediction model

The LASSO model was estimated on the training sample starting with 69 measures, measures shown in Table 2, which were modeled as 82 variables due to the need for some variables to be separated into subsections. The lambda for the model was estimated at 0.02 using 10-fold cross validation. This yielded an 18-variable model, eliminating 64 variables from the model. The estimated coefficients from the model are presented in Table 3. Most statistical packages purposefully do not provide standard errors for LASSO models. Using bootstrapping and the appropriate covariance matrix may not provide reliable standard errors [74, 75]. Because of this, and since accurate prediction is the primary purpose of the model, we do not include confidence intervals for the estimated coefficients and only provide confidence intervals for the predictions of the model.

**Table 2:** Initial set of measures used to train model.

Measures	Range
**Sociodemographic factors (9)**	
Age	0/1
Gender	0/1
Race	0/1
Education	0/1
Marital status	0/1
Having kid(s)	0/1
Employment status	0/1
Self-reported physical health status	0/1
Income	$0 –$200,000
**Adverse childhood events (9)**	
Emotional abuse by parents	0/1
Physical abuse by father	0/1
Physical abuse by mother	0/1
Sexual assault	0/1
Not loved by parents	0/1
Not taken care of by parents	0/1
Lost biological parents because of death or divorce	0/1
Lived with drinker	0/1
Lived with drug addict	0/1
**Life problems (7)**	
Everyday discrimination	9–36
Discrimination (major)	0–11
Current work situation	0–10
Family strain	4–16
Friend strain	4–16
Marital risk	1–9
Current rating of financial situation	0–10
**Major life trauma (14)**	
Ever flunked out of school	0/1
Ever fired from a job	0/1
Ever no job for long time	0/1
Ever parents divorced	0/1
Ever spouse/partner engaged in infidelity	0/1
Ever significant in-law difficulties	0/1
Death(s) in the family in the last 5 years	0/1
Ever lost home to fire/flood/etc	0/1
Ever sexually assaulted	0/1
Ever serious legal difficulties/prison	0/1
Ever jail detention	0/1
Ever bankruptcy declared	0/1
Ever financial loss unrelated to work	0/1
Ever experienced combat	0/1
**Psychological factors (14)**	
General Distress-Depress Symptoms	12–60
General Distress-Anxious Symptoms	11–55
Loss of Interest	8–40
High Positive Affect	14–70
Anxious Arousal	17–105
CES-D	0–60
Kessler K6 (using a 5-point Likert scale (1–5), then divided by 6)	1–5
Anger expression–in	8–32
Anger expression–out	8–32
Anger control	4–16
Trait anger	15–60
Religious/spiritual coping	2–8
Problem focused coping	12–48
Emotion focused coping	12–48
**Personality traits (6)**	
Conscientiousness	1–4
Agreeableness	1–4
Neuroticism	1–4
Openness	1–4
Extraversion	1–4
Somatic amplification	1–4
**Clinical factors (8)**	
Body Mass Index	17.18–54.91
Sleep quality	1–4
Number of vehicle injuries	0–4
Number of broken bones	0–20
Number of surgeries	0–10
Number of head injuries	0–3
Number of joint injuries	0–3
Low pain tolerance	1–4
**Baseline pain status (2)**	
Having acute pain	0/1
Having chronic pain	0/1

**Table 3:** Estimated LASSO coefficients–FUTUREPAIN predictive model.

Variable	Coefficient
Current chronic pain status	4.08
BIOLOGICAL FACTORS	
Number of surgeries	0.56
Number of vehicle accidents with injuries	1.87
Number of head injuries	0.30
Number of joint injuries	1.10
Body mass index	0.22
Sleep quality	3.57
Excellent health	-1.24
Fair health	6.01
PSYCHOLOGICAL FACTORS	
Kessler K6	7.40
Loss of Interest	1.03
Anxious arousal	0.48
Religious coping	0.59
SOCIAL FACTORS	
Not in workforce	1.10
Parental abuse	4.12
Death in family	2.43
Lost home	4.69
Financial or property loss	3.58

Some items could legitimately be placed in more than one category.

The model yielded an AUROC of 0.85 (95% Confidence Interval (CI): 0.79, 0.91) when using out-of-sample predictions from the testing sample. The model also produced an AUROC of 0.81 (95% CI: 0.73, 0.89) in the testing sample when those with moderate-to-severe chronic pain at baseline were removed. The optimal cut-point in the testing sample at which sensitivity (0.88) and, specificity (0.75) was maximized was 16 (95% CI: 13, 18) where Youden’s *J* index was 0.632. The optimal cut-point did not change when observations indicating moderate-to-severe chronic pain at baseline were removed. The resulting final questionnaire and scoring algorithm can be found in the S1 File. An online questionnaire with automatic scoring can be accessed at www.futurepain.org.

## Discussion

More than 30% of US adults are afflicted by chronic pain and this prevalence is expected to increase with population aging [3]. The economic burden of chronic pain is considerable, approximately $560–635 billion annually [2].

We developed a questionnaire that evaluates the probability of an adult experiencing moderate-to-severe chronic pain in 7-to-10 years. We examined sociodemographic factors, adverse childhood events, life problems, major life traumas, psychological factors, personality traits, clinical factors, and baseline pain status based on national longitudinal data of a middle-aged US population.

A machine learning approach to variable selection, LASSO, was used to reduce 82 variables to a more practical 18. This was transformed into a final questionnaire comprised of 17 questions (the number of questions does not exactly equal the number of variables because of the manner in which the variables were constructed from the questions) or 47 sub-items, requiring approximately 6 minutes to complete (see S1 File). A score of 16 or higher indicates that a person has a strong possibility of experiencing moderate-to-severe chronic pain in 7–10 years and would benefit from preventive treatment.

Our study is novel in that it tested multiple categories of the determinants of chronic pain simultaneously. Specifically, we combined sociodemographic factors, adverse childhood events, life problems, major life traumas, psychological factors, personality traits, clinical factors, and baseline pain status as candidate predictors of chronic pain. Moreover, this is the first attempt to estimate the probability of having moderate-to-severe chronic pain 7–10 years into the future.

Regarding the elements contained in the final questionnaire produced by this model, it is important to note that the methodology used here aims only to predict, not to determine the causal relationship between any given measure and future pain status. In other words, while particular items in the final questionnaire have been found to be causally related to pain status in other studies (e.g., the K6 has been found to predict both the initiation and cessation of pain [47, 76]), the specific items included in the final questionnaire can only be said to be strongly associated with future pain status; they should not be construed as causally related to future pain status on the basis the predictive model alone. Determining causal relationships with observational data requires alternative methodologies [47, 76].

Thus, the fact that some elements are included in the questionnaire while others are omitted does not at all imply that the omitted elements are unimportant. Many elements that have been shown to be associated with pain in the literature but do not appear in the final questionnaire may simply occur earlier in the pain development pathway (e.g., daily discrimination and lifetime discrimination work through psychological distress to cause chronic pain [47]) or they may be strongly correlated with those elements included in the final questionnaire, but not quite as strongly correlated with future pain as the elements included in the final questionnaire. Elements not included in the questionnaire are thus still highly relevant to our understanding both of the development and prevention of chronic pain.

### Future directions and current clinical value

The FUTUREPAIN instrument can be used both to perform research on chronic pain and to inform treatment decisions in clinical situations. With regard to research, to determine rigorously the value of the FUTUREPAIN instrument, future longitudinal studies examining the effectiveness of psychological treatment for chronic pain would benefit from incorporating the FUTUREPAIN instrument to determine whether individuals vary in their response to psychological treatment for chronic pain by the various FUTUREPAIN subscores: biological, psychological, and social. We hypothesize that individuals with relatively higher psychological and/or social subscores will be the most likely to benefit from psychological treatment for chronic pain. Such research would allow us to determine rigorously whether the FUTUREPAIN instrument can effectively distinguish between those individuals who would be more likely to benefit from psychological interventions and those who would be less likely to benefit from psychological interventions.

However, such research is not essential for the FUTUREPAIN instrument to be useful in determining whether current patients experiencing chronic pain may be more or less likely to benefit from psychological interventions for chronic pain. Under the following restrictive assumptions, the FUTUREPAIN instrument is likely to strongly indicate that a given chronic pain patient may benefit from psychological interventions for chronic pain: (1) the FUTUREPAIN score is greater than the cut-point, and (2) the biological subscore is only based on the following characteristics: zero surgeries, zero vehicle accidents with injuries, zero head injuries, zero joint injuries, normal BMI (18.5 to 24.9), very good sleep quality (less than 2), and very good health or excellent health. In other words, if the FUTUREPAIN biological factor subscore only describes a healthy set of biological characteristics, this implies that the combined psychological factors and social factors subscores alone are driving the high FUTUREPAIN score. In such a situation, when combined with the clinical judgement of the relevant clinician, the relevant clinician may determine that it is reasonable to assume that psychological interventions for chronic pain are likely to be of benefit for a particular patient.

### Limitations

Our study is not without limitations. The major limitation concerns the nature of predictive models. Predictive models such as the one produced here can never predict with 100% accuracy due to the inability to include or even ascertain every possible relevant variable that may influence future chronic pain status, the inherent limitations of statistical modeling, and most importantly, the complexity of the pain process. Because of this, some misclassification will necessarily occur.

More specific limitations include that fact that about 99% of our sample consists of middle-aged individuals, ranging from 35 to 83. The relative lack of other age groups may limit the generalizability of our study results. Second, variables related to adverse childhood events and major life traumas may be subject to recall bias since this information is obtained retrospectively.

## Conclusion

Currently, the management of chronic pain is performed among patients already suffering from chronic pain. Management is still largely based on improving pathophysiological risk factors and reducing pain intensity via opioid analgesics and other medications. The model we present in this study will help clinicians determine which of their patients are at high risk of experiencing moderate-to-severe chronic pain in the future. It can also help identify individuals who are at the high risk of developing chronic pain even before pain starts, in addition to identifying those whose chronic pain is likely to continue. This is the first tool to achieve these types of predictions using a single instrument.

Early interventions that aim to address the multidimensional risk factors of chronic pain could prevent many people from suffering from chronic pain in the future and the related physical and mental disabilities that accompany chronic pain. Such early intervention may also reduce the economic burden of chronic pain, including overtreatment [77]. Avoiding the physical, emotional, and economic burdens of pain are the goal of the FUTUREPAIN project.

## Supporting information

S1 File (DOCX)
