# Supplementary material for: The FUTUREPAIN study: Validating a questionnaire to predict the probability of having chronic pain 7-10 years into the future
Source: PLoS One. 2020 Aug 20;15(8):e0237508. doi: 10.1371/journal.pone.0237508 (PMC7440636; doi:10.1371/journal.pone.0237508)
Supplement: S1 File — (DOCX) [file pone.0237508.s001.docx]

**FUTUREPAIN Questionnaire and Scoring Algorithm**

1. In general, would you say your physical health is excellent, very good, good, fair, or poor? (Choose one)

| Poor | Fair | Good | Very good | Excellent |
| --- | --- | --- | --- | --- |
| 0 | 1 | 0 | 0 | 1 |

2. What about your current employment situation -- are you working now for pay, self-employed, looking for work, temporarily laid off, retired, a homemaker, a full-time or part-time student, or something else?

| Employed  (e.g. working now, self-employed) | Unemployed  (e.g. looking for work, temporarily laid off) | Not in workforce  (e.g. retired, homemaker, full- or part-time student, maternity or sick leave, permanently disabled, others) |
| --- | --- | --- |
| 0 | 0 | 1 |

3a. During your childhood, how often did either your mother or the woman who raised you, do any of the following things to you? Insulted you or swore at you; sulked or refused to talk to you; stomped out of the room; did or said something to spite you; threatened to hit you; smashed or kicked something in anger. (Choose one)

| Never | Rarely | Sometimes | Often |
| --- | --- | --- | --- |
| 1 | 2 | 3 | 4 |

3b. During your childhood, how often did either your father or the man who raised you, do any of the following things to you? Insulted you or swore at you; sulked or refused to talk to you; stomped out of the room; did or said something to spite you; threatened to hit you; smashed or kicked something in anger. (Choose one)

| Never | Rarely | Sometimes | Often |
| --- | --- | --- | --- |
| 1 | 2 | 3 | 4 |

|  |  | Yes | No |
| --- | --- | --- | --- |
| 4. | Have any of your parents, siblings, or children died during the past 5 years? | 1 | 0 |
| 5. | Have you ever lost your home to fire, flood, natural disaster, etc. ? | 1 | 0 |
| 6. | Have you ever suffered a financial or property loss unrelated to work? | 1 | 0 |
| 7. | Do you have chronic pain, that is do you have pain that persists beyond the time of normal healing and has lasted from anywhere from a few months to many years? | 1 | 0 |

8. How much have you felt or experienced things this way during the past week, including today? (Choose one answer for each row)

|  |  | Not at all | A little bit | Moderately | Quite a bit | Extremely |
| --- | --- | --- | --- | --- | --- | --- |
| A. | Felt unattractive | 1 | 2 | 3 | 4 | 5 |
| B. | Felt withdrawn from other people | 1 | 2 | 3 | 4 | 5 |
| C. | Felt really slowed down | 1 | 2 | 3 | 4 | 5 |
| D. | Felt really bored | 1 | 2 | 3 | 4 | 5 |
| E. | Felt like it took extra effort get started | 1 | 2 | 3 | 4 | 5 |
| F. | Felt like nothing was very enjoyable | 1 | 2 | 3 | 4 | 5 |
| G. | Felt like there wasn’t anything interesting or fun to do | 1 | 2 | 3 | 4 | 5 |
| H. | Thought about death or suicide | 1 | 2 | 3 | 4 | 5 |

9. How much have you felt or experienced things this way during the past week, including today. (Choose one answer for each row)

|  |  | Not at all | A little bit | Moderately | Quite a bit | Extremely |
| --- | --- | --- | --- | --- | --- | --- |
| A. | Startled easily | 1 | 2 | 3 | 4 | 5 |
| B. | Hands were shaky | 1 | 2 | 3 | 4 | 5 |
| C. | Was short of breath | 1 | 2 | 3 | 4 | 5 |
| D. | Felt faint | 1 | 2 | 3 | 4 | 5 |
| E. | Had hot or cold spells | 1 | 2 | 3 | 4 | 5 |
| F. | Hands were cold or sweaty | 1 | 2 | 3 | 4 | 5 |
| G. | Was trembling or shaking | 1 | 2 | 3 | 4 | 5 |
| H. | Had trouble swallowing | 1 | 2 | 3 | 4 | 5 |
| I. | Felt dizzy or lightheaded | 1 | 2 | 3 | 4 | 5 |
| J. | Had pain in my chest | 1 | 2 | 3 | 4 | 5 |
| K. | Felt like I was choking | 1 | 2 | 3 | 4 | 5 |
| L. | Muscles twitched or trembled | 1 | 2 | 3 | 4 | 5 |
| M | Had a very dry mouth | 1 | 2 | 3 | 4 | 5 |
| N. | Was afraid I was going to die | 1 | 2 | 3 | 4 | 5 |
| O. | Heart was racing or pounding | 1 | 2 | 3 | 4 | 5 |
| P | Felt numbness or tingling in body | 1 | 2 | 3 | 4 | 5 |
| Q | Had to urinate frequently | 1 | 2 | 3 | 4 | 5 |

10. During the past 30 days, how much of the time did you feel… (Choose one answer for each row)

|  |  | None of the time | A little of time | Some of the time | Most of the time | All the time |
| --- | --- | --- | --- | --- | --- | --- |
| A. | So sad nothing could cheer you up? | 1 | 2 | 3 | 4 | 5 |
| B. | Nervous? | 1 | 2 | 3 | 4 | 5 |
| C. | Restless or fidgety? | 1 | 2 | 3 | 4 | 5 |
| D. | Hopeless? | 1 | 2 | 3 | 4 | 5 |
| E. | That everything was an effort? | 1 | 2 | 3 | 4 | 5 |
| F. | Worthless? | 1 | 2 | 3 | 4 | 5 |

11a. When you have problems or difficulties in your family, work, or personal life, how often do you seek comfort through religious or spiritual means such as praying, meditating, attending a religious or spiritual service, or talking to a religious or spiritual advisor? (Choose one)

| Never | Rarely | Sometimes | Often |
| --- | --- | --- | --- |
| 1 | 2 | 3 | 4 |

11b. When you have decisions to make in your daily life, how often do you ask yourself what your religious or spiritual beliefs suggest you should do? (Choose one)

| Never | Rarely | Sometimes | Often |
| --- | --- | --- | --- |
| 1 | 2 | 3 | 4 |

12. What is your height and weight (Body Mass Index)?

Height____________ (inches to 1 decimal)

Weight______________ (lbs. to 1 decimal)

13. During the past month, how would you rate your sleep quality overall? (Choose one)

| Very bad | Fairly bad | Fairly good | Very good |
| --- | --- | --- | --- |
| 4 | 3 | 2 | 1 |

14. How many times have you had surgery in your life? (Any type) ____________ times

# 15. How many times have you injured in a motor vehicle accident? ____________ times

# 16. How many times have you had any type of joint injury? ____________ times

# 17. How many times have you had any type of head injury? ____________ times

**FUTUREPAIN Questionnaire Scoring Algorithm**

| **Question** | **Domain** | **Coefficient** |
| --- | --- | --- |
| Q1 | Excellent Health = 1 | -1.24 |
|  | Fair Health = 1 | 6.01 |
| Q2 | Employment status = 1 | 1.10 |
| Q3 | Parental abuse = 1 if 3a $\geq3 AND/O$R 3b $\geq3$ | 4.12 |
| Q4 | Death in family = 1 | 2.43 |
| Q5 | Lost home = 1 | 4.69 |
| Q6 | Financial or property loss = 1 | 3.58 |
| Q7 | Chronic pain status = 1 | 4.08 |
| Q8 | Loss of Interest = sum of all items | 1.03 |
| Q9 | Anxious Arousal = sum of all items | 0.48 |
| Q10 | Kessler K6 = sum of all items and then divide by 6 | 7.40 |
| Q11 | Religious Coping = sum of 11a and 11b | 0.59 |
| Q12 | BMI (weight in lbs x .45)/(height in inches x 0.025)^2 | 0.22 |
| Q13 | Sleep Quality = answer | 3.57 |
| Q14 | Number of Surgeries | 0.56 |
| Q15 | Number of Vehicle Accidents with Injuries | 1.87 |
| Q16 | Number of Joint Injuries | 1.10 |
| Q17 | Number of Head Injuries | 0.30 |

Instructions: Multiply the answer to each question by the coefficient, sum them, and then subtract 44.14. Individuals with scores of 16 or greater are candidates for treatment.
